# A Night-Time Monitoring System (eNightLog) to Prevent Elderly Wandering in Hostels: A Three-Month Field Study

**DOI:** 10.3390/ijerph19042103

**Published:** 2022-02-13

**Authors:** James Chung-Wai Cheung, Eric Wing-Cheung Tam, Alex Hing-Yin Mak, Tim Tin-Chun Chan, Yong-Ping Zheng

**Affiliations:** 1 Department of Biomedical Engineering, Faculty of Engineering, The Hong Kong Polytechnic University, Hong Kong 999077, China; yongping.zheng@polyu.edu.hk; 2 Research Institute for Smart Ageing, The Hong Kong Polytechnic University, Hong Kong 999077, China; 3 Jockey Club Smart Ageing Hub, Department of Biomedical Engineering, Faculty of Engineering, The Hong Kong Polytechnic University, Hong Kong 999077, China; eric.tam@polyu.edu.hk (E.W.-C.T.); alexmak.mak@polyu.edu.hk (A.H.-Y.M.); tctimchan@polyu.edu.hk (T.T.-C.C.)

**Keywords:** elderly, dementia, wandering, night monitoring, bed exiting, virtual constraint, ultrawideband radar, remote sensing, elderly care hostel, nursing home

## Abstract

Older people are increasingly dependent on others to support their daily activities due to geriatric symptoms such as dementia. Some of them stay in long-term care facilities. Elderly people with night wandering behaviour may lose their way, leading to a significant risk of injuries. The eNightLog system was developed to monitor the night-time bedside activities of older people in order to help them cope with this issue. It comprises a 3D time-of-flight near-infrared sensor and an ultra-wideband sensor for detecting human presence and to determine postures without a video camera. A threshold-based algorithm was developed to classify different activities, such as leaving the bed. The system is able to send alarm messages to caregivers if an elderly user performs undesirable activities. In this study, 17 sets of eNightLog systems were installed in an elderly hostel with 17 beds in 9 bedrooms. During the three-month field test, 26 older people with different periods of stay were included in the study. The accuracy, sensitivity and specificity of detecting non-assisted bed-leaving events was 99.8%, 100%, and 99.6%, respectively. There were only three false alarms out of 2762 bed-exiting events. Our results demonstrated that the eNightLog system is sufficiently accurate to be applied in the hostel environment. Machine learning with instance segmentation and online learning will enable the system to be used for widely different environments and people, with improvements to be made in future studies.

## 1. Introduction

The World Health Organization (WHO) has estimated that the global population over 60 years old will continuously grow from 900 million in 2015 to 2 billion by 2050 [1]. Various health issues are emerging with the ageing population. Health issues including falls, dementia, delirium, pressure ulcers, incontinence and frailty are commonly found in elderly people. These issues are collectively known as geriatric syndromes, which are highly prevalent. Older age, cognitive impairment, functional impairment and impaired mobility are four major risk factors for geriatric syndromes [2]. 

Dementia is one of the most concerning issues among the geriatric syndromes. With over 46 million people living with dementia worldwide today, it is recognized as the world’s next epidemic. The population with dementia is increasing sharply with the rapidly ageing global population. The number of people with dementia will reach 131.5 million by 2050. Dementia has also been associated with huge health and social care and medical costs. The total cost worldwide of dementia is expected to reach 2 trillion USD by 2030, which would be equivalent to the world’s 18th largest economy [3,4].

The prevalence of dementia rapidly increases with advancing age. It has been reported that the rate increases from 1.4% in those who are 65–69 years old to 23.6% in those over 85 years old [5], peaking at 58% among individuals over 94 years old [6]. Hence, the prevalence of dementia will increase significantly with the rapid population ageing expected in developed countries and regions, including Hong Kong, which holds the longest average life expectancy in the world. The projected number of people aged 60 or above with dementia in Hong Kong is projected to increase by 222%, from 103,433 in 2009 to 332,688 in 2039 [7]. 

Furthermore, the costs of long-term health and social care will dramatically increase, causing a considerable economic burden. It has been reported that there will be approximately three million people aged 60 or above by 2039, which would be around one-third of the total population in Hong Kong [8]. With people with dementia increasing and reductions in global workforces, the question of how elderly care services can be maintained has arisen. Approximately 70% of people diagnosed with dementia suffer from Alzheimer’s disease (AD). Vascular dementia, dementia with Lewy bodies, and a group of diseases that contribute to frontotemporal deterioration are other common forms of dementia [9]. 

Dementia often develops slowly with non-obvious early signs similar to other illnesses. The progression of dementia from the normal ageing brain often starts with mild cognitive impairment (MCI) as the intermediate stage [10]. It is also difficult to distinguish mild grade dementia from normal ageing. A review found that the prevalence of MCI varies largely among international studies from around 3% to 42% [11]. It has also been reported that the annual rate of progression from MCI to dementia ranges from 10% to 15% [12,13]. The progression of dementia varies between people and also between different types of the disease. The symptoms progress slowly over several years in most cases, because cognitive loss observed in patients with AD and pathological changes caused by brain degeneration commence at least 10–20 years prior to dementia onset [14]. Neuropsychiatric symptoms, including psychosis, agitation, apathy, depression, bruxism and sleep disturbances could be hallmarks of AD [15,16]. 

People with dementia may find that their abilities to remember, think, make decisions and speak worsen as dementia progresses, considerably affecting daily functioning. Patients’ increasing needs for assistance in daily activities commonly include dressing, toileting and eating. Their behaviour may change, with a decrease in motivation, depression and demoralization being commonly reported symptoms. The development of anxieties or phobias is quite common among patients. Anger or agitation is another common behaviour problem in the later stages of dementia. Challenging behaviours, including aggression, can be provoked [17,18].

Wandering is a frequent behavioural disorder and one of the most important and challenging management aspects for dementia patients. It is also one of the most common symptoms of dementia, and has been reported as an exhausting behaviour for caregivers [19] that leads to care home admission. Wandering in the elderly is an acutely distressing problem worldwide. In a previous study, more than two-thirds of the participating caregivers regarded wandering as a risk of fall or missing patients [20]. However, the aetiology of wandering is unresolved and poorly understood. Wandering is generally recognized as aimless ambulation in living facilities. Although wandering is common clinically, its definition has not yet been well-established. One of the reasons for this is that wandering is sometimes conflated with agitated behaviour [21]. It is a broad term, encompassing a diverse set of behaviours found in the literature [21]. There are approximately two key features among various definitions of wandering: the elderly being cognitively impaired and moving through space. It has been agreed that wandering means aimless or disoriented ambulation throughout a facility, with common patterns encompassing lapping, pacing, or random ambulation [19,22]. However, it can also occur in people who need assistive devices or a wheelchair for ambulation [21]. From a physiological perspective, wandering could be driven by circadian rhythm disturbances and dysfunction of spatial perception and memory [21,23]. From a psychosocial viewpoint, internal discomfort may cause wandering behaviour, which is aroused by personal needs, such as toileting, calling for help, and searching for a person, and aggravated by external factors, such as noise [24]. 

The prevalence of wandering has varied among studies, with 17.4% of community-residing seniors [25] and 63% in community dwellings having been reported to have exhibited wandering behaviour [26]. More than 60% of people with different levels of dementia will develop wandering behaviour [27]. Wandering can lead to severe injuries, patients going missing, and fatal accidents. Up to 50% of wanderers have suffered from severe to fatal injury. Subsequently, significant care and economic burdens are caused by this behaviour [28]. For instance, hostel caregivers experienced emotional distress that led to potential civil tort claims and penalties to employers [29]. The diminishing of language skills, weight loss, victimization and premature mortality could be accelerated in at-risk wanderers [30]. For example, a study revealed that nursing home residents who suffered from wandering had a 2.7 times higher risk of fall than the non-wanderers over three months [31]. Wanderers also reported a two-fold risk of fracture compared to non-wanderers [32].

Wandering-associated falls are one of the leading causes of morbidity and mortality. The frequency of falls increases with advancing age. The fall incidence in a hospital was 2.5 per 1000 patient days, and more than 60% of the fall cases were elderly people [33]. Around 5% to 10% of falls have serious outcomes, including laceration, bone fracture or head injury [34]. Dementia is also an independent risk factor for falls. Elderly people with dementia have been found to be two to three times more likely to fall [35], and it has been reported that they find it more challenging to recover [36]. Clearly, the costs for the elderly with dementia in medical care and rehabilitation after a fall are enormous. With these wandering-associated adverse outcomes in elderly people with dementia, there is a pragmatic demand to develop relevant systems or management to mitigate the stress of caregivers and protect the elderly [37]. Physical exercise can combat the reduction of physical functioning, and thus prevent falls caused by wandering [38]. However, this requires additional resources to support it, and most hostels or hospitals have resolved to apply restraints.

**Figure 1:**
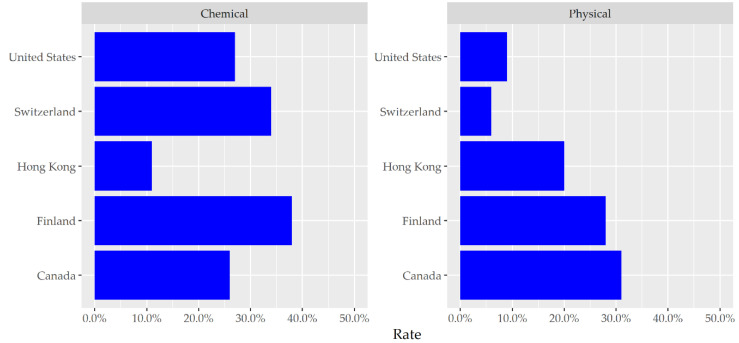
Physical and chemical restraint usage rates in countries/regions [39].

The rate of restraint usage has been reported to be different across the globe (Figure 1). It varies more than fivefold across countries, with averages of 6% in Switzerland, 9% in the United States, 20% in Hong Kong, 28% in Finland, and over 31% in Canada being reported. The rate of chemical restraint usage has been reported to be similarly wide, ranging from 11% in Hong Kong to 26–27% in Canada and the United States, 34% in Switzerland, and nearly 38% in Finland [39]. Surprisingly, the use of restraints has increased despite ethical issues. An 11-year longitudinal study (2005 to 2015) revealed that physical and chemical restraint applications increased from 57.9% to 75.7% and from 15.9% to 21.78%, respectively [40]. In Japan, a high rate of 44.5% of physical restraint use was also reported in a national cross-sectional study [41].

Physical restraint is defined as using a device attached or adjacent to the target body from which the person cannot unravel themselves, in order to restrict the target freedom of movement [42]. Bilateral bedside rails, trunk restraint, chair-boards (which consist of a chair with a fixed tray table), boxing gloves and straitjackets are common types of physical restraint in the health sector [43,44]. Physical restraints can also protect the safety of the elderly during the use of medical equipment, such as mechanical ventilators [45], control behaviours such as aggression and restlessness, and promote positional support [45,46,47]. Physical restraints are more frequently applied on patients with dementia or any form of cognitive impairment to prevent injuries to the patient or other people [48]. Chemical restraints can be psychotropic medications, such as antipsychotic, antianxiety, or hypnotic agents. They serve the same purpose as physical restraint in clinical settings [40]. In Hong Kong, a higher frequency of physical restraint usage but lower chemical restraint usage compared to other developed countries or regions has been reported [40].

The use of physical restraints is controversial. Restrained individuals cannot satisfy basic personal needs, such as stretching limbs, using the bathroom, and drinking water. It would be against one’s conscience and affects mental well-being. Furthermore, strangulation and entrapment under restraints can result in more severe injuries than falls. People who have experienced restraints have reported negative feelings and psychological trauma, such as helplessness, depression, fear, anger, anxiety, demoralization and the loss of dignity [49]. Additionally, negative reactions, including screaming, fighting and extreme agitation can be triggered, causing potential injuries to the person or his/her caregivers [50]. There are other negative health-associated consequences, including respiratory complications, poor nutrition, urinary incontinence and constipation, reduced cardiovascular endurance, increased agitation and dependence in activities of daily living, increased mortality, impaired muscle strength, poor balance, decubitus ulcers and bruises [51]. 

With evidence pointing to significant adverse consequences and the ineffectiveness of restraints [52,53], the Joint Commission and the US Food and Drug Administration (FDA) have revised guidelines and emphasized the minimization of physical restraints in patient care settings [54]. Similar to physical restraints, chemical restraints also have adverse effects. For instance, antipsychotic drugs have many side effects, such as drowsiness, gait disturbance and chest infections. These drugs may also impair attention to diet, leading to an increased risk of hospitalization. Furthermore, the risk of adverse cerebrovascular effects of antipsychotic drug use, such as stroke, has been found to approximately double, with 1.7-times higher mortality reported over two years [55].

With sensors and remote sensing technologies continuing to emerge, virtual restraint has been increasingly accepted as a substitute for physical and chemical restraints. Surveillance cameras, infrared detectors, pressure sensors, location devices, wearable devices, and related telecare products have been frequently used as virtual restraints in elderly hostels. The pressure and infrared sensors have been commonly used but not integrated into bedding and need to be configured before use. These systems can work standalone or connected to a central monitoring system in the sector. However, the functions and positions of the sensors have to be checked from time to time. 

In summary, physical and chemical restraints have resulted in negative consequences for the elderly, while the current virtual restraints are inconvenient to use. Some studies have developed non-invasive methods, but these lack evaluations in practicability with field studies. In view of this, a night-time monitoring system, the eNightLog system, has been developed to monitor elderly night-time activities and prevent wandering, using non-contact sensors that can overcome some limitations of pressure mats and infrared fences. The performance of the eNightLog system was proven to be superior to the integrated pressure mat and infrared fence system in a laboratory test simulating nursing home settings in our previous work [56]. However, the system has not been evaluated with elderly people with dementia living in a hostel setting. Therefore, this field study aimed to assess the performance of eNightLog systems in an elderly care home in multiple bedroom settings. The contribution of this study lies in its potential to support wandering prevention in hostels as a regular measure.

## 2. Materials and Methods

This field study is built on the work of our previous research which evaluated the eNightLog system in a controlled setting only [56]. This field study tested 17 sets of the systems in one hostel over 3 months.

Here, we give an overview of the materials and methods chapter. The method starts with the introduction of subject recruitment and the protocol design of the field study. Secondly, the field setup section describes the environment of the field study, the location and configuration of our system, including network servers and alarm feedback. The third section on system architecture sets out the different functional components and their linkage within the system. The fourth section explains how the system classified bed-exiting and non-bed-exiting events, while the last section states the metrics used for evaluating the classification performance. 

### 2.1. Subject Recruitment and Protocol

We recruited 7 male and 19 female participants aged 56 to 113 years (male: M = 83.5, SD = 12.97; female: M = 85.1, SD = 12.19) and with heights ranging from 139 to 173 cm (male: M = 163.8, SD = 7.48; female: M = 153.9, SD = 6.35). The participants had different periods of stay and were circulated in the hostel. All participants were diagnosed with dementia without any form of physical disabilities.

All participants were required to leave their rooms before 7 a.m. by themselves (non-assisted bed-exiting) or assisted by caregivers (assisted bed-exiting), and were not allowed to return to their rooms before 8 p.m. At 10 p.m., all participants were sent to bed, supervised by caregivers, with the lights off. The eNightlog systems carried out surveillance from 10 p.m. to 7 a.m. Neither training nor instructions were provided to the participants. All bed-exiting events were manually examined and categorized into non-assisted and assisted bed exiting. Assisted bed exiting means that an elderly person acquired help from staff to leave their bed. 

### 2.2. Field Setup

**Figure 2:**
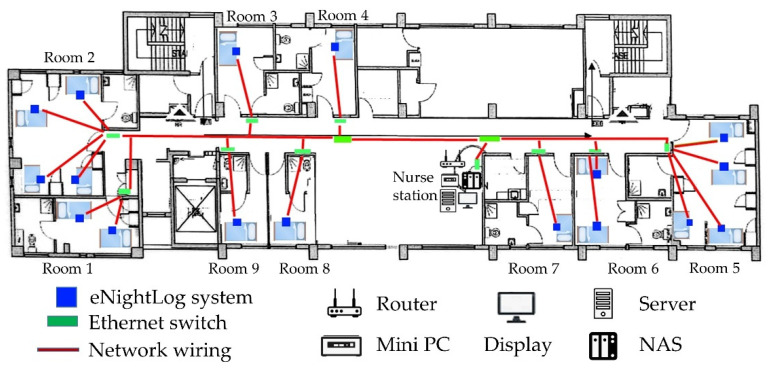
Floorplan, network connections, server and eNightLog system locations on the field site.

Seventeen eNightLog systems were built and installed in a hostel with five single rooms, two double rooms and two quadruple rooms. The hostel was an elderly dementia care and attention home. Access was restricted to authorized caregivers using a password-locked door in order to prevent residents from escaping. The residents were invited to participate in the study with consent from their guardians (Figure 2). All systems were networked and linked to the nurse station server and network storage. The server also linked a monitor and two mobile phones for notification and tracking.

The status and alarm were sent in a network message attachment with the status and timestamp to corresponding servers for data storage, using network-attached storage (NAS) and visualization in a local computer network. 

**Figure 3:**
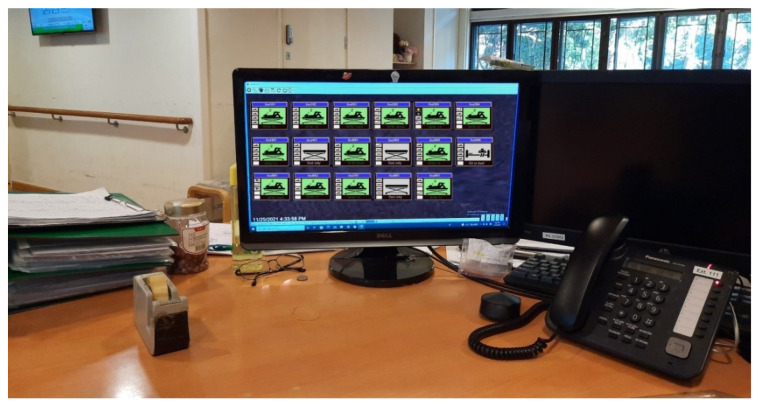
Monitor at the nurse station showing the subject status and providing notification.

All 17 systems were linked to the local server to monitor residents continuously. A server application was created and installed in the local server to simultaneously monitor and display the status of elderly people at the nurse station (Figure 3). The silhouette formatted images were captured at one frame per second, stored in each eNightLog system and relocated to the NAS weekly according to the balance among the storage size, I/O speed, and network performance. Since camcorder video was not allowed due to privacy, a video was generated using silhouette formatted images captured as the ground truth, with the frame rate being one frame per second for each bed every night. 

**Figure 4:**
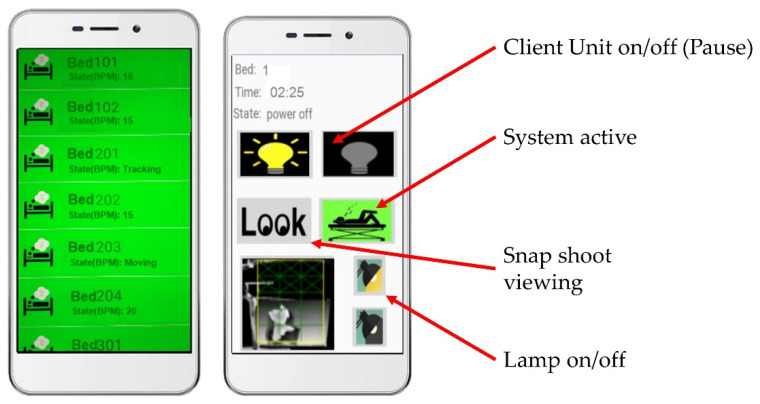
Alarm notification of eNightLog system and room control via a mobile device and applications.

Each eNightLog system was linked to an Internet-of-Things (IoT) device, Raspberry Pi 3 Model B (Raspberry Pi foundation, Cambridge, UK), and the remote accessing software, Teamviewer (TeamViewer GmbH, Göppingen, Germany), for remote maintenance and configuration. In addition, two mobile phones with eNightLog-system-supporting apps were provided to the authorized caregivers in order for them to receive alarm messages (Figure 4). A data analysis panel was developed for data visualization. It also allowed manual labelling and the storage of patient status with the generated video. 

### 2.3. System Development and Architectures

The eNightlog system was developed based on a remote sensing approach in order to circumvent any physical contact and usage setup and overcome the hurdles of setup, staffing, and suspicion of being monitored.

Its main components consisted of two sensors and a mini personal computer (Intel NUC i5-8BEH, Intel Corp., Santa Clara, CA, USA) with a supporting algorithm and customized software. An infrared 3D time-of-flight (ToF) sensor (Kinect V2 sensor, Microsoft, Redmond, WA, USA), in which a built-in RGB camera was concealed to protect privacy, and an Ultra-Wideband Impulse Radar (UWB-IR) sensor (Xethru, Novelda, Norway), acted as the main component. The UWB-IR detected the subject movement and presence and measured the respiration rate. The software was written with C# using Microsoft Visual Studio version 2019 (Microsoft, Redmond, WA, USA), which supported visualization, image processing, device and network connectivity, posture and position classification, alarm notification and system configuration.

**Figure 5:**
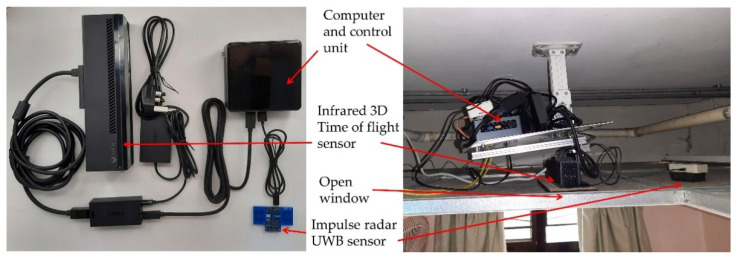
eNight System component and setup inside ceiling.

The eNightLog system was mounted on the ceiling to ensure privacy and maximize coverage. In addition, this minimized the chance of overlapping objects, particularly in the presence of multiple subjects or objects. There was a narrow window opening for the eNightLog systems to allow the trans-receiving of infrared light (Figure 5). No opening windows were made for the UWB-IR sensor, as the radar electromagnetic signal can penetrate the non-metallic ceiling tile without significant signal attenuation.

**Figure 6:**
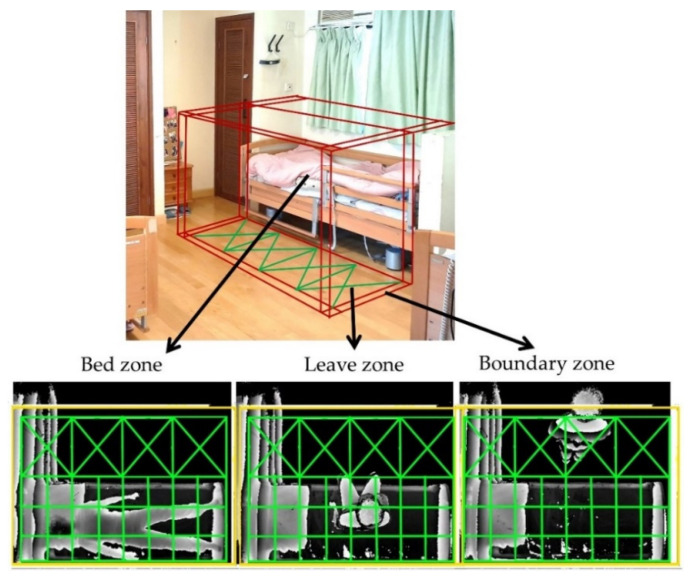
The monitoring zones of eNightLog system (bed zone, leave zone, boundary zone).

The 3D ToF sensor produced depth images. They can be considered as quasi-3D because a depth image was acquired from a single viewpoint, and the information on the overlapping objects in the rear position would have been lost if the front object blocked the line of sight. In order to transform coordinates from scene depth to height, image data were mapped into a new coordinate system. Initially, the images captured by the 3D ToF sensor had a resolution of 512 pixels (X) by 424 pixels (Y), with the depth values representing the horizontal distance between the sensor and body. Subsequently, the Y pixels and depth values were recast to image length and depth height, respectively, because of the mounting setting. The depth value was converted into greyscale by normalizing the range from the captured closest object to the farthest object and was subsequently revamped into a solid silhouette with contour shading (Figure 5) to make the resultant image unidentifiable for privacy. The 3D ToF sensors were mounted 230 to 270 cm above the ground. Three monitor zones, namely the bed zone, leave zone and boundary zone, were established for each bed using virtual boundaries as a virtual fence (Figure 6) set by the eNightLog software. These virtual fences were exploited to support the location classification and to detect, using a previously developed algorithm [56], the moment the subject crossed the boundary.

### 2.4. Classification Method

**Figure 7:**
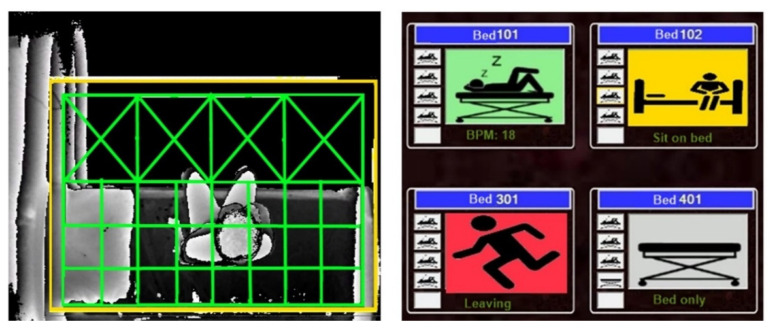
Depth image captured a participant sitting by the bed (**left**) and user interface indicating different status and alarms (**right**)—red (exit), yellow (sit in zone), green (lying), grey (empty).

The developed algorithm used the decision tree approach [57] with information from 3DToF and UWB-IR and pre-set boundaries for classifying positions and postures. The posture status included sleeping, lying, sitting, standing, bed-exiting, sitting on the edge, and others. Some states were used to indicate transient conditions and were not displayed. The system allowed different pre-set alarm levels corresponding to the position and posture of the subject. As shown in Figure 7, a yellow alarm indicated that the subject was staying at the boundary between the bed and the leaving zone, which could represent the subject either sitting on the edge of the bed or standing by the bed and potentially intending to exit the bed soon. A red alarm noted that the subjects had departed the leaving zone via the pre-set boundary zone. The subjects’ exiting the leaving zone might be recognized as wandering behaviour or other activities with potential risks. In general, the activities happening near the bed (leave zone) would only raise the alarm to the yellow (first level) alarm. In this study, only the bed-exiting state was involved in the performance evaluation.

### 2.5. Evaluation Method

The evaluation of the system targeted the correct classification of bed-exiting events with the confusion matrix listing true or false and positive or negative [58]. Sensitivity (or true positive rate) indicates the proportion of participants who had a bed-exiting event reported by the system and who confirmed the event as being correct, while specificity (or true negative rate) indicates the proportion of participants who had a non-bed-exiting event reported and confirmed the event as correct. Accuracy is the fraction of correct classification reports from the system over the total number of reports. Precision (or positive predictive value) is the probability of really having a bed-exiting event if the system reported the event. The correctness of the event reports was verified by researchers watching and labelling the generated video. The reports of the system on bed-exiting events were marked in all timestamps.

## 3. Results

**Figure 8:**
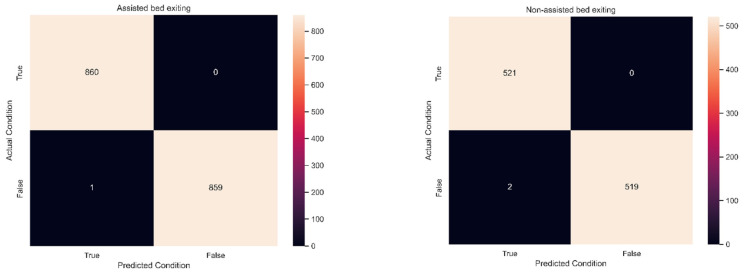
Confusion matrix for assisted bed existing (**left**) and non-assisted bed exiting (**right**) events.

The system classified bed-exiting events based on the algorithm-based functions of the eNightLog, as detailed in Section 2.4. The ground truth bed-exiting events were annotated on the generated video manually throughout the 3-month study period. There were 1042 counts of non-assisted bed-exiting events and 1720 counts of assisted bed-exiting events (Figure 8). Table 1 shows an overview of the eNightLog performance. The results showed that the system had excellent performance in all evaluation metrics. The accuracy, precision, sensitivity, and specificity of the eNightLog under the non-assisted and assisted bed-exiting events were almost identical. Accuracy, precision, and specificity were slightly higher in the assisted bed-exiting cases than in the non-assisted bed-exiting cases. The sensitivity was the same in both bed-exiting events.

## 4. Discussion

In this study, the eNightLog system was validated with excellent performance and showed only 3 false alarms out of 2762 bed-exiting events over three months. The system revealed its capability of performing wandering surveillance in a practical environment and of potentially replacing existing products such as pressure sensors.

Pressure sensors in the form of mats are commonly used to control bed-exiting, but these require constant maintenance, including cleaning, disinfection and repositioning before usage [59], causing an extra workload for caregivers. Furthermore, most of these pressure sensors feature audible alarm functions, which could cause distress to other residents in hostels, especially elderly people with dementia. It is therefore crucial to minimize unnecessary alarm caused by false alarms [60]. Cities with land scarcity, such as Hong Kong, may have irregular alignment of beds for private hostels. In this study, as the beds were not aligned on the same side but arranged to maximize separation, elderly individuals allocated to the inner bed needed to pass through the bed zone of their roommates. This arrangement could likely trigger a false alarm of the infrared fence. The single-bed, two-bed and four-bed rooms with different layouts were included in this study to evaluate the system performance when facing scenarios prone to false alarms. This study revealed that the eNightLog system could maintain good performance regardless of bedroom layouts for bed-exiting events in practice. 

Hostels with multiple beds in the same room may induce a higher chance of false alarms because of the frequency of participants and caregivers ambulating across the systems. It was expected that the accuracy performance in the field test could have been slightly inferior to that in the laboratory due to uncontrollable environmental and human factors. Surprisingly, the performance of the system in the field study was slightly better than that in our laboratory testing (Table 2) [56]. The reason for this could be that the hostel residents moved slower than the young subjects in our laboratory test, and particularly for assisted events. The frame rate of the generated video could also have affected the accuracy performance. Although the eNightLog might not have a sufficient frame rate and image quality to capture and recognize all bed-exiting events and visitor activities in detail, it can still produce a generated video to serve as a ground truth reference, which was important here, since multiple subjects and objects may have interfered with the identification of targeted individuals. 

In the sleeping rooms of hostels, video cameras are prohibited. We used raw data from the 3D ToF sensor to produce an image in the form of a solid silhouette with contour shading with caregiver comments in order to produce the ground reference (Figure 2). The resultant silhouette image might enable rough identification through body features such as height and body size. The advantage of this feature was that the facial and clothing features were masked, and person identification could be encrypted. Nevertheless, three elderly people refused to participate in our study because of privacy concerns, while similar reports on technological surveillance were also found [61]. We plan to reconstruct a skeleton avatar using the images and discard the image records in our next work. 

Since our detection strategy greatly relied on the field of vision of the 3D ToF sensor, the camera/sensors were placed in a position to maximize camera coverage and minimize blockade. However, items inside the zone coverage could potentially change frequently and affect the field of view. For instance, large items in the coverage, such as a wheelchair, walking aids or furniture, could misguide the system. In this case, the left items can be ruled out by a time mask, particularly for stationary objects. The system can also alert the caregiver to remove or park the items properly. In addition, a wearable beacon using wireless communication, or an infrared LED indicator can be given to residents and caregivers to identify their presence better.

At present, the UWB-IR sensor in the system identifies the presence of individuals by searching for the presence of a breathing rate and cross-checking with other sensors. Furthermore, this sensor can scan through walls and furniture and does not contribute to wearing discomfort or privacy issues, since there is no image [62].

Table 3 compares our system with existing systems using different sensors or approaches. The eNightLog system outperformed the systems in the other studies in all metrics. The specificity for the systems using the pressure mat, infrared fence and multiple pressure mat, thermal array with an ultrasonic sensor, and eNightLog were approximately the same. This means that false alarm rates were low in their studies. However, all these previous studies were conducted in the laboratory setting. The thermal array and RFID are relatively new sensors for application in this field. However, the thermal array may interfere with the environment and residual heat signature, while RFID could be poor in finding position information. Regardless, we may consider integrating these sensors in our future studies. 

The infrared fence bed-exiting sensor and pressure mat for identifying bed-exiting events has shown good performance and low cost, making it a good alternative. However, there is increasing concern for its practical usage because the U.S. Food and Drug Administration (FDA) has revealed many reports of false alarms and failures to issue alarms accurately. Many caregivers use both an infrared fence and pressure mat to improve sensitivity and minimize false alarms, though this is yet to be evaluated [70]. Moreover, elderly people are aware of the device and may develop negative feelings of being monitored. 

Recently, position and posture change alarm products have been increasingly accepted as tools to manage wandering behaviours, including wearable beacon transmitters, chair and bed sensor pads, bedside alarm mats, alarms clipped to clothing, seatbelt alarms, and infrared bed-exiting alarms. These devices are virtual restraints that restrict people by alerting caregivers to stop wanderers. One problem is that these virtual restraint devices trigger audible alarms. As a result, residents might be afraid of the nuisance to other residents and staff; thus, they may be self-restricting, sensitive to sound, and result in adverse outcomes, such as loss of dignity, decreased mobility, bowel and bladder incontinence, sleep disturbances, confusion, fear, agitation and anxiety [39].

In this study, the strength of the eNightLog system was that it alerted caregivers but was not noticed by the elderly residents, thus reducing nuisance and psychological distress. Alerting messages were sent to the caregivers via mobile devices. However, caregivers commented that they needed to check their mobile devices frequently during work. We will improve our system by using wall-hang flashlights and auto-voice broadcasts in the nurse station. The system features and performance can be further improved with wearable devices and deep learning techniques.

Wearable devices can provide posture information to recognize fall events for elderly residents through accelerometers [71]. They are also widely used and accepted by the public and have a range of measurement functions to support health surveillance and care planning, such as medicine reminders, the measurement of core and skin body temperature, blood oximetry, heart rate, respiration rate, blood pressure, mood, urine volume, and are backed by industrial leaders. Integrating wearables to the eNightLog can enhance its function to auto-adjust the monitoring zones and accommodate the dynamics of residents, caregivers, and visitors in hostels. Furthermore, the integrated system can facilitate features for smart healthcare, such as fall detection, fall-out and rollover on beds, and musculoskeletal, posture and sleep quality assessment, etc. [72,73]. In the future, machine learning techniques could be applied to these data to predict bed-exiting or wandering behaviour based on their in-bed postures and movement, sleep problems, and other physiological measurements. We also anticipate that our future system will be able to automatically control the on/off and colour of light sources to ease sleeplessness or other sleep-related problems. However, bedding, particularly quilts or blankets, can substantially impede the system performance. Recently, a deep learning approach has overcome this problem and paved a way to understand sleep posture [74]. Alternatively, a few UWB-IR sensors could be arranged in a grid to perform data fusion in order to produce a low-resolution image for estimating the posture and even monitoring wandering events. The eNightLog exploited a PC as a core controller linked with sensors, a network hub and an IoT device, making the internal architecture rather complicated. Despite this, it can link to other developed accessories such as smart diapers, toilet sensors, etc. The PC was costly and not designed for continuous functioning, making the system maintenance a challenging issue. A higher performance single board computer should be used to convert current system coding into a corresponding language platform.

### Limitations and Application of Study

A limitation of the eNightLog system was the stringent clearance between the system and the bed. The minimum distance should be 2.2 m, from system to bed, to have sufficient coverage over all three zones, as shown in Figure 2. The purpose of the leaving zone was to create a buffer area for the residents to inhabit near to the bed but to remain cautious of potential bed-exiting events. Therefore, the size of the buffering leaving region should be large enough for daily living with assistive tools. In fact, an IR-3DToF sensor with a wider angle could resolve this limitation. However, there are few options that could satisfy the current application.

The scenario of multiple beds in a single room is typical in hostels due to scarce resources and space. In Hong Kong, the living environment is very dense, and four or more residents often share a small room, which creates significant challenges to monitoring systems. In this study, our system endeavoured to accommodate different room settings, including different bed alignments and the number of beds in a single room. However, the problem is further complicated with the non-aligned placement of beds, double-deck beds, and hanging beds, which is very common in low-income communities. It would be more economical and feasible to enable monitoring of multiple individuals in the same room. Indeed, this would involve a complete redesign of the algorithm, which requires a component to track and identify individuals. Machine learning with instantaneous segmentation and online training could provide the foundation for this new algorithm in our future system [58]. 

## 5. Conclusions

The ageing population is the most significant emerging healthcare burden globally, especially elderly people with dementia and wandering behaviour. In care homes or hospitals, to control wandering behaviour or walkaways for safety, physical and chemical restraints have been used, but these have been accused of poor effectiveness and ethical violations, while virtual restraints have often launched false alarms, causing a huge nuisance to both the elderly and caregivers. The night-time monitoring system (eNightLog) developed in this study demonstrated excellent accuracy in recognizing bed-exiting events in a practical environment. It could serve as an effective surveillance system to ease the burden on caregivers and protect the privacy of the elderly. 

## 6. Patents

A Chinese invention patent (No. 201710706823.X) has been filed for the eNightLog, and it has been publicized in: https://patents.google.com/patent/CN109394223A/en?oq=cn109394223A (accessed on 5 December 2021).

## Figures and Tables

**Table 1:** Bed-exiting event recognition and evaluation results.

Setting and Outcome	Non-Assisted Bed Exiting	Assisted Bed Exiting
Total number of events detected	1042	1720
True Positive (TP)	521	860
True Negative (TN)	519	859
False Positive (FP)	2	1
False Negative (FN)	0	0
Accuracy	99.8%	99.9%
Precision	99.6%	99.9%
Sensitivity	100%	100%
Specificity	99.6%	99.9%

**Table 2:** Performance of eNightLog in different bedroom layout settings.

Study	Our Previous Work [56]			This Study	
Setting	Laboratory Test			Field Test	
	Single Bed	Single Bed	Double Bed	Multiple Beds & Rooms	
Assisted	No			No	Yes
System	IFPM	eNightLog	eNightLog	eNightLog	eNightLog
No. of bed-exiting events	1800	1800	9000	1042	1720
Accuracy	85.9%	99.0%	98.8%	99.8%	99.9%
Precision	78.6%	99.2%	97.8%	99.6%	99.9%
Sensitivity	98.9%	98.8%	99.9%	100%	100%
Specificity	73.0%	99.2%	97.8%	99.6%	99.9%

IFPM: infrared fence and pressure mat.

**Table 3:** Comparison with different studies in bedside event recognition.

Sensor(s)	Source	Accuracy	Precision	Sensitivity	Specificity
Infrared fence	[63]	-	-	85.3%	96.2%
Pressure mat	[63]	-	-	90.4%	99.3%
Pressure sensor	[64]	-	-	96%	95.5%
Infrared fence and multiple pressure mats	[63]	-	-	92.3%	99.4%
RFID	[65]	-	-	93.8%	90.8%
Thermal array and ultrasonic sensor	[66]	95.5%	93.8%	71.4%	99.3%
Kinect	[67]	98.8%	-	-	-
Colour Camera	[68]	87.7%	90.6%	83.1%	92.1%
Video	[69]	-	59.4%	97.4%	-
eNightLog (this study)	Assisted	99.9%	99.9%	100%	99.9%
	Non-assisted	99.8%	99.6%	100%	99.6%

## Data Availability

The data presented in this study are available on request from the corresponding author. The data are not publicly available due to the privacy of the subjects.

## References

[1] World Health Organization 10 Facts on Ageing and Health. https://www.who.int/news-room/fact-sheets/detail/10-facts-on-ageing-and-health.

[2] Inouye S.K., Studenski S., Tinetti M.E., Kuchel G.A. (2007). Geriatric Syndromes: Clinical, Research, and Policy Implications of a Core Geriatric Concept: (See Editorial Comments by Dr. William Hazzard on pp 794–796). J. Am. Geriatr. Soc..

[3] Alzheimer’s Disease International World Alzheimer Report 2015. The Global Impact of Dementia: An Analysis of Prevalence, Incidence, Cost and Trends. https://www.alz.co.uk/research/WorldAlzheimerReport2015.pdf.

[4] Alzheimer’s Disease International World Alzheimer Report 2018. The State of the Art of Dementia research: New Frontiers. https://www.statista.com/statistics/471323/global-dementia-economic-impact-forecast/.

[5] Jorm A., Korten A., Henderson A. (1987). The prevalence of dementia: A quantitative integration of the literature. Acta Psychiatr. Scand..

[6] Ebly E.M., Parhad I.M., Hogan D.B., Fung T. (1994). Prevalence and types of dementia in the very old: Results from the Canadian Study of Health and Aging. Neurology.

[7] Yu R., Chau P.H., McGhee S.M., Cheung W.L., Chan K.C., Cheung S.H., Woo J. (2012). Trends in prevalence and mortality of dementia in elderly Hong Kong population: Projections, disease burden, and implications for long-term care. Int. J. Alzheimer’s Dis..

[8] Census and Statistics Department of Hong Kong Hong Kong Population Projections 2010–2039. https://www.statistics.gov.hk/pub/B1120015042010XXXXB0100.pdf.

[9] Barker W.W., Luis C.A., Kashuba A., Luis M., Harwood D.G., Loewenstein D., Waters C., Jimison P., Shepherd E., Sevush S. (2002). Relative frequencies of Alzheimer disease, Lewy body, vascular and frontotemporal dementia, and hippocampal sclerosis in the State of Florida Brain Bank. Alzheimer Dis. Assoc. Disord..

[10] Petersen R.C., Caracciolo B., Brayne C., Gauthier S., Jelic V., Fratiglioni L. (2014). Mild cognitive impairment: A concept in evolution. J. Intern. Med..

[11] Ward A., Arrighi H.M., Michels S., Cedarbaum J.M. (2012). Mild cognitive impairment: Disparity of incidence and prevalence estimates. Alzheimer’s Dement..

[12] Petersen R.C., Doody R., Kurz A., Mohs R.C., Morris J.C., Rabins P.V., Ritchie K., Rossor M., Thal L., Winblad B. (2001). Current concepts in mild cognitive impairment. Arch. Neurol..

[13] Geda Y.E. (2012). Mild cognitive impairment in older adults. Curr. Psychiatry Rep..

[14] Holtzman D.M., Morris J.C., Goate A.M. (2011). Alzheimer’s disease: The challenge of the second century. Sci. Transl. Med..

[15] Lanctôt K.L., Amatniek J., Ancoli-Israel S., Arnold S.E., Ballard C., Cohen-Mansfield J., Ismail Z., Lyketsos C., Miller D.S., Musiek E. (2017). Neuropsychiatric signs and symptoms of Alzheimer’s disease: New treatment paradigms. Alzheimer’s Dement. Transl. Res. Clin. Interv..

[16] Heyat M.B., Akhtar F., Khan M.H., Ullah N., Gul I., Khan H., Lai D. (2021). Detection, Treatment Planning, and Genetic Predisposition of Bruxism: A Systematic Mapping Process and Network Visualization Technique. CNS Neurol. Disord. Drug Targets (Former Curr. Drug Targets—CNS Neurol. Disord.).

[17] World Health Organization (2019). Dementia Fact Sheet. https://www.who.int/en/news-room/fact-sheets/detail/dementia.

[18] Burns A., Iliffe S. (2009). Dementia. BMJ.

[19] Rolland Y., Gillette-Guyonnet S., Nourhashémi F., Andrieu S., Cantet C., Payoux P., Ousset P., Vellas B. (2003). Wandering and Alzheimer’s type disease. Descriptive study. REAL. FR research program on Alzheimer’s disease and management. Rev. De Med. Interne.

[20] Utton D. (2009). The design of housing for people with dementia. J. Care Serv. Manag..

[21] Cipriani G., Lucetti C., Nuti A., Danti S. (2014). Wandering and dementia. Psychogeriatrics.

[22] Teri L., Larson E.B., Reifler B.V. (1988). Behavioral disturbance in dementia of the Alzheimer’s type. J. Am. Geriatr. Soc..

[23] Tetewsky S.J., Duffy C.J. (1999). Visual loss and getting lost in Alzheimer’s disease. Neurology.

[24] Phillips V.L., Diwan S. (2003). The incremental effect of dementia-related problem behaviors on the time to nursing home placement in poor, frail, demented older people. J. Am. Geriatr. Soc..

[25] Klein D.A., Steinberg M., Galik E., Steele C., Sheppard J.M., Warren A., Rosenblatt A., Lyketsos C.G. (1999). Wandering behaviour in community-residing persons with dementia. Int. J. Geriatr. Psychiatry.

[26] Hope T., Tilling K.M., Gedling K., Keene J.M., Cooper S.D., Fairburn C.G. (1994). The Structure of Wandering in Dementia. Int. J. Geriatr. Psychiatry.

[27] Agrawal A.K., Gowda M., Achary U., Gowda G.S., Harbishettar V. (2021). Approach to Management of Wandering in Dementia: Ethical and Legal Issue. Indian J. Psychol. Med..

[28] Alzhemier’s Association Alzhemier’s Disease Facts and Figures. http://www.alz.org/facts/.

[29] Stevenson D.G., Studdert D.M. (2003). Trends—The rise of nursing home litigation: Findings from a national survey of attorneys. Health Aff..

[30] Algase D. (2005). Wandering: Clues to effective management. Geriatr. Aging.

[31] Colombo M., Vitali S., Cairati M., Perelli-Cippo R., Bessi O., Gioia P., Guaita A. (2001). Wanderers: Features, findings, issues. Arch. Gerontol. Geriatr. Suppl..

[32] Wick J.Y., Zanni G.R. (2006). Aimless excursions: Wandering in the elderly. Consult. Pharm..

[33] Yaghoubi S., Gooraji S.A., Habibi M., Torkaman F. (2021). Fall incidence in hospitalized patients and prediction of its risk factors using a weighted Poisson model. J. Public Health.

[34] Rubenstein L.Z., Josephson K.R. (2002). The epidemiology of falls and syncope. Clin. Geriatr. Med..

[35] Kröpelin T.F., Neyens J.C., Halfens R.J., Kempen G.I., Hamers J.P. (2013). Fall determinants in older long-term care residents with dementia: A systematic review. Int. Psychogeriatr..

[36] Shaw F.E. (2002). Falls in cognitive impairment and dementia. Clin. Geriatr. Med..

[37] Nelson A.L. (2007). Evidence-Based Protocols for Managing Wandering Behaviors.

[38] Dionyssiotis Y. (2012). Analyzing the problem of falls among older people. Int. J. Gen. Med..

[39] Feng Z., Hirdes J.P., Smith T.F., Finne-Soveri H., Chi I., Du Pasquier J.N., Gilgen R., Ikegami N., Mor V. (2009). Use of physical restraints and antipsychotic medications in nursing homes: A cross-national study. Int. J. Geriatr. Psychiatry.

[40] Lam K., Kwan J.S.K., Wai Kwan C., Chong A.M.L., Lai C.K.Y., Lou V.W.Q., Leung A.Y.M., Liu J.Y.W., Bai X., Chi I. (2017). Factors Associated with the Trend of Physical and Chemical Restraint Use Among Long-Term Care Facility Residents in Hong Kong: Data From an 11-Year Observational Study. J. Am. Med. Dir. Assoc..

[41] Nakanishi M., Okumura Y., Ogawa A. (2018). Physical restraint to patients with dementia in acute physical care settings: Effect of the financial incentive to acute care hospitals. Int. Psychogeriatr..

[42] Kwok T., Bai X., Chui M.Y.P., Lai C.K.Y., Ho D.W.H., Ho F.K.Y., Woo J. (2012). Effect of Physical Restraint Reduction on Older Patients’ Hospital Length of Stay. J. Am. Med. Dir. Assoc..

[43] Kwok T., Mok F., Chien W.T., Tam E. (2006). Does access to bed-chair pressure sensors reduce physical restraint use in the rehabilitative care setting?. J. Clin. Nurs..

[44] Yan E., Kwok T., Lee D., Tang C. (2009). The prevalence and correlates of the use of restraint and force on hospitalised older people. J. Nurs. Healthc. Chronic. Illn..

[45] Choi E., Song M. (2003). Physical restraint use in a Korean ICU. J. Clin. Nurs..

[46] Capezuti E. (2004). Minimizing the use of restrictive devices in dementia patients at risk for falling. Nurs. Clin. N. Am..

[47] Gallinagh R., Nevin R., Mc Ilroy D., Mitchell F., Campbell L., Ludwick R., McKenna H. (2002). The use of physical restraints as a safety measure in the care of older people in four rehabilitation wards: Findings from an exploratory study. Int. J. Nurs. Stud..

[48] Hofmann H., Hahn S. (2014). Characteristics of nursing home residents and physical restraint: A systematic literature review. J. Clin. Nurs..

[49] Lancaster G.A., Whittington R., Lane S., Riley D., Meehan C. (2008). Does the position of restraint of disturbed psychiatric patients have any association with staff and patient injuries?. J. Psychiatr. Ment. Health. Nurs..

[50] Andrews G.J. (2006). Managing challenging behaviour in dementia. BMJ.

[51] Gastmans C., Milisen K. (2006). Use of physical restraint in nursing homes: Clinical-ethical considerations. J. Med. Ethics.

[52] Robinson L., Hutchings D., Corner L., Beyer F., Dickinson H., Vanoli A., Finch T., Hughes J., Ballard C., May C. (2006). A systematic literature review of the effectiveness of non-pharmacological interventions to prevent wandering in dementia and evaluation of the ethical implications and acceptability of their use. Health Technol. Assess..

[53] Behrman S., Dunn M. (2010). Physical restraint of medical inpatients: Unravelling the red tape. Clin. Ethics.

[54] Dimant J. (2003). Avoiding physical restraints in long-term care facilities. J. Am. Med. Dir. Assoc..

[55] Ooi C.H., Yoon P.S., How C.H., Poon N.Y. (2018). Managing challenging behaviours in dementia. Singap. Med. J..

[56] Cheung J.C.-W., Tam E.W.-C., Mak A.H.-Y., Chan T.T.-C., Lai W.P.-Y., Zheng Y.-P. (2021). Night-time monitoring system (eNightLog) for elderly wandering behavior. Sensors.

[57] Lai D., Heyat M.B.B., Khan F.I., Zhang Y. (2019). Prognosis of sleep bruxism using power spectral density approach applied on EEG signal of both EMG1-EMG2 and ECG1-ECG2 channels. IEEE Access.

[58] Mao Y.-J., Lim H.-J., Ni M., Yan W.-H., Wong D.W.-C., Cheung J.C.-W. (2022). Breast Tumour Classification Using Ultrasound Elastography with Machine Learning: A Systematic Scoping Review. Cancers.

[59] Shinmoto Torres R.L., Visvanathan R., Abbott D., Hill K.D., Ranasinghe D.C. (2017). A battery-less and wireless wearable sensor system for identifying bed and chair exits in a pilot trial in hospitalized older people. PLoS ONE.

[60] Shorr R.I., Chandler A.M., Mion L.C., Waters T.M., Liu M., Daniels M.J., Kessler L.A., Miller S.T. (2012). Effects of an intervention to increase bed alarm use to prevent falls in hospitalized patients: A cluster randomized trial. Ann. Intern. Med..

[61] Demiris G., Hensel B.K., Skubic M., Rantz M. (2008). Senior residents’ perceived need of and preferences for “smart home” sensor technologies. Int. J. Technol. Assess. Health Care..

[62] Cho H.S., Park Y.J. (2018). Detection of Heart Rate through a Wall Using UWB Impulse Radar. J. Healthc. Eng..

[63] Lu C., Huang J., Lan Z., Wang Q. Bed exiting monitoring system with fall detection for the elderly living alone. Proceedings of the 2016 International Conference on Advanced Robotics and Mechatronics (ICARM).

[64] Hilbe J., Schulc E., Linder B., Them C. (2010). Development and alarm threshold evaluation of a side rail integrated sensor technology for the prevention of falls. Int. J. Med. Inf..

[65] Ranasinghe D.C., Shinmoto Torres R.L., Hill K., Visvanathan R. (2014). Low cost and batteryless sensor-enabled radio frequency identification tag based approaches to identify patient bed entry and exit posture transitions. Gait Posture.

[66] Asbjørn D., Jim T. (2017). Recognizing Bedside Events Using Thermal and Ultrasonic Readings. Sensors.

[67] Ni B., Nguyen C.D., Moulin P. RGBD-camera based get-up event detection for hospital fall prevention. Proceedings of the 2012 IEEE International Conference on Acoustics, Speech and Signal Processing (ICASSP).

[68] Bu F., Lin Q., Allebach J. (2021). Bed exit detection network (BED Net) for patients bed-exit monitoring based on color camera images. Electron. Imaging.

[69] Jones K.J., Haynatzki G., Sabalka L. (2021). Evaluation of automated video monitoring to decrease the risk of unattended bed exits in small rural hospitals. J. Patient Saf..

[70] Capezuti E., Brush B.L., Lane S., Rabinowitz H.U., Secic M. (2009). Bed-exit alarm effectiveness. Arch. Geronto.l Geriatr..

[71] Cheung C.-W.J., Chan W.-H.R., Chiu M.-W., Law S.-Y., Lee T.-H., Zheng Y.-P. A three-month study of fall and physical activity levels of intellectual disability using a transfer belt-based motion recording sensor. Proceedings of the 6th World Congress of Biomechanics (WCB 2010).

[72] Garn H., Kohn B., Dittrich K., Wiesmeyr C., Kloesch G., Stepansky R., Wimmer M., Ipsiroglu O., Grossegger D., Kemethofer M. 3D detection of periodic limb movements in sleep. Proceedings of the 2016 38th Annual International Conference of the IEEE Engineering in Medicine and Biology Society.

[73] Wong D.W.-C., Wang Y., Lin J., Tan Q., Chen T.L.-W., Zhang M. (2019). Sleeping mattress determinants and evaluation: A biomechanical review and critique. PeerJ.

[74] Tam A.Y.-C., So B.P.-H., Chan T.T.-C., Cheung A.K.-Y., Wong D.W.-C., Cheung J.C.-W. (2021). A Blanket Accommodative Sleep Posture Classification System Using an Infrared Depth Camera: A Deep Learning Approach with Synthetic Augmentation of Blanket Conditions. Sensors.

